# Molecular Evolution of the Rice Blast Resistance Gene *Pi-ta* in Invasive Weedy Rice in the USA

**DOI:** 10.1371/journal.pone.0026260

**Published:** 2011-10-17

**Authors:** Seonghee Lee, Yulin Jia, Melissa Jia, David R. Gealy, Kenneth M. Olsen, Ana L. Caicedo

**Affiliations:** 1 Rice Research Extension Center, University of Arkansas, Stuttgart, Arkansas, United States of America; 2 The Samuel Roberts Noble Foundation, Ardmore, Oklahoma, United States of America; 3 Dale Bumpers National Rice Research Center, Agricultural Research Service, United States Department of Agriculture, Stuttgart, Arkansas, United States of America; 4 Department of Biology, Washington University, St. Louis, Missouri, United States of America; 5 Department of Biology, University of Massachusetts, Amherst, Massachusetts, United States of America; Seoul National University, Republic of Korea

## Abstract

The *Pi-ta* gene in rice has been effectively used to control rice blast disease caused by *Magnaporthe oryzae* worldwide. Despite a number of studies that reported the *Pi-ta* gene in domesticated rice and wild species, little is known about how the *Pi-ta* gene has evolved in US weedy rice, a major weed of rice. To investigate the genome organization of the *Pi-ta* gene in weedy rice and its relationship to gene flow between cultivated and weedy rice in the US, we analyzed nucleotide sequence variation at the *Pi-ta* gene and its surrounding 2 Mb region in 156 weedy, domesticated and wild rice relatives. We found that the region at and around the *Pi-ta* gene shows very low genetic diversity in US weedy rice. The patterns of molecular diversity in weeds are more similar to cultivated rice (*indica* and *aus*), which have never been cultivated in the US, rather than the wild rice species, *Oryza rufipogon*. In addition, the resistant *Pi-ta* allele (*Pi-ta*) found in the majority of US weedy rice belongs to the weedy group strawhull awnless (SH), suggesting a single source of origin for *Pi-ta*. Weeds with *Pi-ta* were resistant to two *M. oryzae* races, IC17 and IB49, except for three accessions, suggesting that component(s) required for the *Pi-ta* mediated resistance may be missing in these accessions. Signatures of flanking sequences of the *Pi-ta* gene and SSR markers on chromosome 12 suggest that the susceptible *pi-ta* allele (*pi-ta*), not *Pi-ta*, has been introgressed from cultivated to weedy rice by out-crossing.

## Introduction

Weedy rice is commonly found in cultivated rice fields and shares traits with both cultivated and wild rice. Weedy rice causes significant economic loss in rice producing areas of the Southern US and is the same species as cultivated rice (*O. sativa* L.) [1]. In the US, common weedy rice, strawhull awnless (SH), blackhull awned (BHA), and brownhull awned (BR) strains are believed to have been introduced to the US as seed contaminants during crop importation and subsequently evolved through natural outcrossing [2], [3], [4], [5], [6]. However, little is known about where US weedy rice has been originated from and how weedy population has been evolved in US rice growing area with other cultivated rice over 100 years. Recently, a few studies have been reported for more precise understanding the genomic diversity of US weedy rice and molecular evolution of important traits (shattering, red pigmentation, and phenol reaction locus *Ph1*) in weedy rice [2], [3], [5], [6]. In these studies, the research groups generated a large DNA sequence dataset for the target locus by sequencing at and flanking region of the locus. This approach for genome-wide survey of nucleotide polymorphisms has enhanced our understanding of the molecular evolution and evolutionary origin of the gene in US weedy rice.

Crop-weed hybridization poses a serious threat as a mechanism for enhancing the ecological fitness of weedy rice in rice growing areas, especially where direct seeding is used for production [7], [8], [9], [10]. New weedy rice ecotypes that originated through spontaneous natural hybridization with commercial rice have been found in commercial fields in the US. Gealy *et al* [11] predicted that new hybrids were derived from crop-weed hybridization and hybridization among the genetically distinct SH and BHA weed strains in nature [11]. Gross *et al*. [3] also reported that some phenol reaction *Phr1* haplotypes in US weeds may be derived from crop-weed hybridization. Genome-wide patterns of single nucleotide polymorphism (SNP) variation in US weedy rice, cultivated, and wild *Oryza* species have demonstrated further evidence of crop-weed hybridization [5]. The movement of an engineered alien herbicide resistance gene from transgenic rice to weedy rice was estimated under field conditions in Korea and China [12]. These studies support the notion that gene flow between cultivated and weedy rice species can frequently occur in regions where weedy rice is commonly found. To date, there is no information available for gene flow of any known disease resistance gene between cultivated and weedy rice through natural hybridization.

Rice blast disease is one of the most threatening diseases for rice production worldwide. The *Pi-ta* gene in rice has been effectively used to manage blast disease in the Southern US and worldwide [13], [14], [15]. Three *indica* landrace cultivars Tadukan (Philippines), Tetep (Vietnam), and Te Qing (China) are known as the sources of resistant *Pi-ta* allele (*Pi-ta*) worldwide [15], [16], [17]. Tadukan is the common donor of *Pi-ta* for various Asian *japonica* cultivars, but the presence of *Pi-ta* in certain *japonica* cultivars (ex. Japanese cultivar, Yashiro-mochi) was derived from unknown *indica* parent(s) different from Tadukan [17], suggesting multiple origins of *Pi-ta* in *japonica* cultivars. Katy was the first US cultivar to contain *Pi-ta* from Tetep and it has become the principal donor for subsequent development of numerous elite US resistant cultivars [14], [15], [18], [19].

The *Pi-ta* gene belongs to a NBS-LRR type of plant resistance (*R*) gene encoding a nucleotide binding site (NBS) and leucine-rich domains (LRD) [20], [21]. The LRD of Pi-ta was demonstrated to directly interact with a putative product of *AVR-Pita* in triggering disease resistance [21]. The structural diversity of the Pi-ta protein and variants in cultivated and wild rice groups of AA genome *Oryza* species has been investigated [14], [22], [23], [24]. These studies revealed that the LRD domain is highly conserved among cultivated and wild species of rice. In contrast, an excess of amino acid substitutions over neutral expectations was observed in the NBS region of both groups, consistent with positive selection. Within the LRD, a functional amino acid polymorphism (Serine to Alanine) at position 918 of Pi-ta is found in all cultivated resistant rice and wild relatives. In addition, two recent studies determined the relationship between the size of the *Pi-ta* genomic block introgression and blast disease resistance; and at least 5 Mb of the *Pi-ta* genomic block with another required component *Ptr(t)* is found in resistant cultivars containing *Pi-ta* [23], [25], [26].

Studying genome organization of the *Pi-ta* gene in weedy rice will not only help us gain more insight into the molecular mechanisms of blast resistance genes, but also will benefit the development of strategies to manage weedy species of rice in commercial rice fields. The objectives of the present study were to evaluate: i) natural variation at/around the *Pi-ta* region in the US weedy rice population; ii) the origin of the *Pi-ta* alleles in US weedy rice, iii) essential genomic region for *Pi-ta*-mediated resistance and iv) gene flow at the *Pi-ta* gene between cultivated and weedy rice.

## Results

### Nucleotide variation at and around the *Pi-ta* gene in US weedy rice

**Figure 1:**
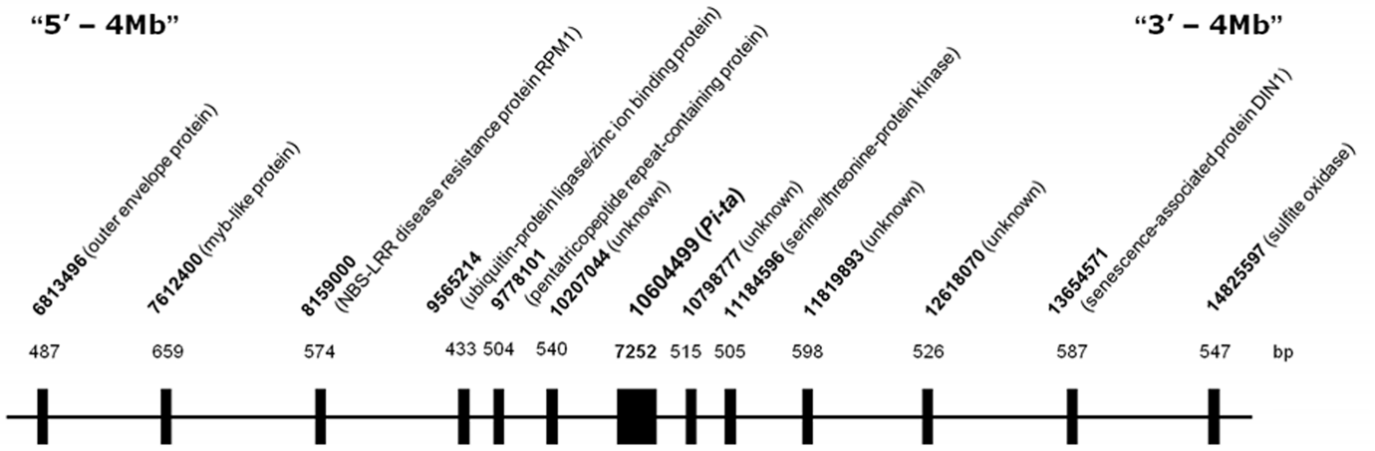
Schematic map showing location of the sequenced region. Numbers in base pair (bp) on the top of gene symbols indicate the length of sequences used for the analysis. The 7252 bp of the *Pi-ta* gene including 5′ leader, coding region and 3′ trailer sequences and 12 gene fragments within 8 megabases were shown.

To determine nucleotide variation at the *Pi-ta* gene in US weedy rice, we sequenced 7252 bp of the *Pi-ta* gene including 5′ leader and 3′ trailer regions (Figure 1). In general, most nucleotide variations were observed in the intron and the 5′ and 3′ noncoding region of the *Pi-ta* gene (Figure S1). The nucleotide variation for coding region in US weedy rice was substantially lower (θ_π_ = 0.00067) than that observed in non-coding region (θ_π_ = 0.00161) of the *Pi-ta* gene (Table 1). Within the coding region, the level of nucleotide diversity was substantially low in exon 2, which contains leucin rice domain (LRD), in all rice accessions. In US weedy rice, θ_π_ and θ_w_ for exon 1 were 0.00114 and 0.00139, while both values were 0.00043 and 0.00047 in exon 2 respectively (Table 1). Most amino acid polymorphisms occurred in the N-terminal and LRD region of the Pi-ta protein (Table 2). Higher values of synonymous nucleotide diversity (π_syn_ = 0.00080) than nonsynonymous nucleotide diversity (π_non_ = 0.00063) were found across the coding region of the *Pi-ta* gene in weedy rice. The average value of π _non_ /π _syn_ for coding region was 0.420 (Table 2), indicating purifying selection in weedy rice in the US.

**Table 1:** Nucleotide polymorphism and divergence of different regions of the *Pi-ta* gene including 5′ and 3′ region in US weedy rice and cultivated rice groups of *O. sativa*, and *O. rufipogon*.

		*Oryza rufipogon*	US weedy	US cultivar	*japonica* 1	*indica*	*aus*	*aromatic*
5′ leader	θ_π_ per site	0.01314	0.00077	0.00225	0.00191	0.00192	0.00065	0.00065
	θ_w_ per site	0.04689	0.00153	0.00180	0.00145	0.00265	0.00086	0.00065
Exon 1	θ_π_ per site	0.00328	0.00114	0.00275	0.00267	0.00159	0.00036	0.00071
	θ_w_ per site	0.00891	0.00139	0.00197	0.00186	0.00158	0.00047	0.00071
Intron	θ_π_ per site	0.00453	0.00262	0.00301	0.00299	0.00317	0.00158	0.00360
	θ_w_ per site	0.01138	0.00219	0.00229	0.00185	0.00280	0.00207	0.00360
Exon 2	θ_π_ per site	0.00115	0.00043	0.00075	0.00068	0.00059	0.00000	0.00000
	θ_w_ per site	0.00375	0.00047	0.00067	0.00054	0.00065	0.00000	0.00000
3′ trailer	θ_π_ per site	0.00484	0.00256	0.00445	0.00412	0.00363	0.00232	0.00464
	θ_w_ per site	0.01064	0.00322	0.00348	0.00281	0.00475	0.00305	0.00464
Coding	θ_π_ per site	0.00186	0.00067	0.00142	0.00135	0.00092	0.00012	0.00024
	θ_w_ per site	0.00547	0.00078	0.00111	0.00099	0.00096	0.00016	0.00024
Noncoding	θ_π_ per site	0.00477	0.00161	0.00270	0.00253	0.00227	0.00110	0.00219
	θ_w_ per site	0.00607	0.00185	0.00210	0.00173	0.00261	0.00144	0.00219

1 US cultivar (*tropical japonica*) samples were not included in *japonica* group (*temperate* and *tropical japonica*) for this analysis.

θ_π_: the average pairwise nucleotide diversity, θ_w_: Watterson's estimator nucleotide variation.

**Table 2:** Analysis of the molecular diversity at the Pi-ta variant in US weedy rice.

Gene and protein region	S^1^	π _syn_ 2	π _non_ 3	π _non_ /π _syn_
*O. sativa* US weedy rice (n = 58)^4^				
Coding	10	0.00120	0.00050	0.420
52849 coding to NBS	6	0.00307	0.00123	0.401
NBS	0	0.00000	0.00000	n.a.^5^
NBS to LRD	0	0.00000	0.00000	n.a.
LRD	4	0.00121	0.00063	0.519
Intron	15	0.00265	n.a.	n.a.
Entire gene	25	0.00080	0.00063	0.787

1 Indicates number of polymorphic sites.

2 Indicates nucleotide diversity at synonymous site.

3 Indicates nucleotide diversity at nonsynonymous site.

4 The number in parentheses indicates the sample size.

5 Not applied.

Strawhull awnless (SH) and Blackhull awned (BHA) are the two major groups of weedy rice in the US. Among them, SH showed the lowest diversity values (θ*_π_* = 0.00007 and θ*_w_* = 0.00015), while θ*_π_* and θ*_w_* for BHA were similar with that in all US weedy accessions (Table 3). Tajima's *D* values for US weedy rice and BHA did not significantly deviate from neutrality (*D* = −0.44628) and (*D* = 0.81700) respectively. This is similar to *indica* (*D* = −0.55670) and *temperate japonica* (*D* = −0.61237), suggesting balancing selection. However, Tajima's *D* for a weedy group SH was significantly negative (*D* = −1.51378), consistent with selective sweep similar to what was observed in *aus* and *O. rufipogon*. As expected, the total and silent site (synonymous and non-coding) nucleotide diversities (π) were low for US weedy rice accessions (θ*_π_* = 0.00161 and θ*_w_* = 0.00185) compared with that in cultivated rice (θ*_π_* = 0.00274 and θ*_w_* = 0.00231) and wild species *O. rufipogon* (θ*_π_* = 0.00477 and θ*_w_* = 0.01358) (Table 3).

**Table 3:** Nucleotide variation of the *Pi-ta* gene in US weedy rice and cultivated rice groups of *O. sativa*, and *O. rufipogon*.^1^

						Cultivated *Oryza sativa*						
		*Oryza rufipogon* (29)	All US weedy (58) 2	SH (24)	BHA (24)	All cultivated *O. sativa* (53)	*indica* (17)	*aus* (6)	*temperate japonica* (4)	*tropical japonica* (8)	US cultivar (15)	*aromatic* (3)
θ_π_ per site	All	0.00477	0.00161	0.00007	0.00173	0.00274	0.00227	0.00110	0.00007	0.00210	0.00270	0.00219
	Silent	0.00607	0.00191	0.00009	0.00208	0.00319	0.00271	0.00149	0.00000	0.00238	0.00306	0.00297
θ_w_ per site	All	0.01358	0.00185	0.00015	0.00143	0.00231	0.00261	0.00144	0.00007	0.00264	0.00210	0.00219
	Silent	0.01476	0.00220	0.00021	0.00166	0.00277	0.00318	0.00195	0.00000	0.00298	0.00238	0.00297
Tajima's D	All	−2.57275	−0.44628	−1.51378	0.81700	0.64571	−0.55670	−1.50278	−0.61237	0.32292	1.22499	N/A^3^
	Silent	−2.57491	−0.43615	−1.51378	0.95891	0.51707	−0.61807	−1.50052	N/A	0.37353	1.21707	N/A
# of polymorphic sites (all)		375	62	4	39	78	64	24	1	50	50	24
# of polymorphic sites (silent)		330	52	4	32	66	55	23	0	40	40	23

1 Total number of sites within 7252 bp was analyzed using DnaSP 5.10. θ*_π_* indicates the average pairwise nucleotide diversity, θ*_w_* indicates Watterson's estimator nucleotide variation. The number of accession for each group analyzed was marked in parenthesis.

2 BR and MIX weedy accessions and accessions of *O. barthii, O. glaberrima, O. glumapatula, O. nivara*, and *O. meridionalis*, were excluded for this analysis.

3 N /A, tests were not performed because of lack of polymorphism.

To determine if similar nucleotide sequence diversity exists around the *Pi-ta* gene, six flanking fragments from 9.6 Mb to 11.8 Mb in all rice accessions were sequenced (Figure 1). From 137 single nucleotide polymorphisms (SNPs), the value of nucleotide diversity across the *Pi-ta* gene and flanking regions was very low in US weedy rice (π = 0.00152 and θ*_w_* = 0.00159), compared to that in cultivated and wild *Oryza* species (Table 4). The weedy group SH showed the lowest value of nucleotide diversity (θ*_w_* = 0.00025), but the diversity level of BHA (θ*_w_* = 0.00141) was similar to that in US weedy rice. This result agrees with the diversity patterns at the *Pi-ta* gene shown in Table 2.

**Table 4:** Nucleotide diversity of flanking region (2 Mb) of the *Pi-ta* gene in US weedy rice and cultivated rice groups of *O. sativa*, and *O. rufipogon.*

Rice group^1^	N^2^	Nt^3^	S^4^	θ*_w_* 5	π^6^
*O. rufipogon*	28	3524	63	0.00459	0.00355
All US weedy rice	58	3524	26	0.00159	0.00152
SH	24	3524	8	0.00061	0.00025
BHA	24	3524	16	0.00122	0.00141
All cultivated *O. sativa*	53	3524	33	0.00202	0.00214
US culivars (*tropical japonica*)	15	3524	16	0.00140	0.00169
*indica*	17	3524	29	0.00243	0.00255
*aus*	6	3524	6	0.00075	0.00074
*aromatic*	3	3524	6	0.00114	0.00114
*temperate japonica*	4	3524	4	0.00062	0.00057
*tropical japonica*	8	3524	10	0.00109	0.00089
Total		3524	137	0.00690	0.00378

1 BR and MIX of weedy rice and US cultivars were excluded from *tropical japonica* samples in this analysis.

2 Indicates sample size.

3 Indicates number of sites, sequence align gaps were not included for analyzing polymorphisms of all sequences.

4 Indicates number of polymorphic (segregating) site.

5 Indicates silent nucleotide polymorphism

6 Indicates nucleotide diversity based on silent site.

**Figure 2:**
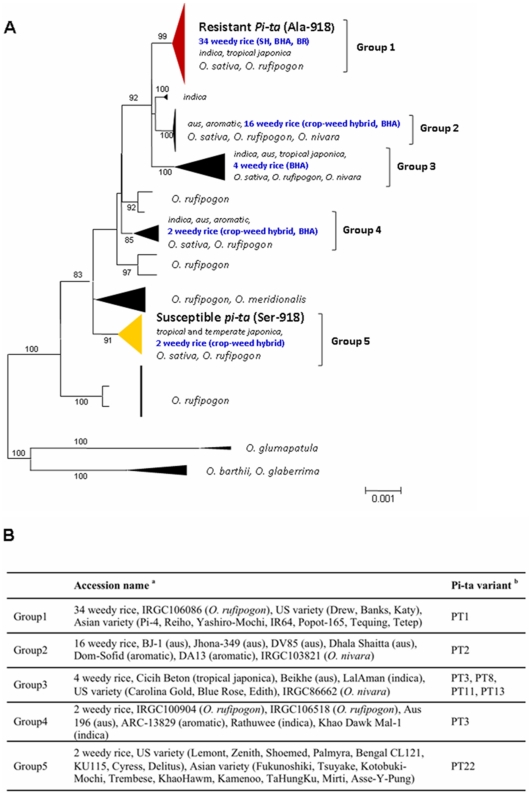
Phylogenetic tree showing groups of US weedy rice among rice populations. (A) An evolutionary tree with genetic distance and group name shown. Red group indicates accession with *Pi-ta* (Ala-918). Yellow group indicates *pi-ta* (Ser-918). Dark black group indicates *pi-ta* (Ser-918 with more amino acid polymorphisms at different positions). (B) Rice accessions classified in each group in the panel A.^ a^ Group name for weedy rice accessions described in Table S2. ^b^ The Pi-ta variants [20].

To group the US weedy rice, a neighbor-joining analysis was performed based on 11 Kb sequence data (excluding alignment gaps) including six gene fragments nearby the *Pi-ta* gene in diverse *Oryza* accessions (Figure 2). The phylogenetic analysis showed that US weedy rice belonged to five groups containing eight different Pi-ta variants. *Pi-ta* (Ala-918) was found in BHA, BR and most of SH, which were grouped with *indica*, *tropical japonica* (possessing *indica*-derived *Pi-ta*) and *O. rufipogon* (Figure 2). Other BHA accessions without *Pi-ta* were grouped with *aus* and *aromatic* landraces. In a previous study, it was shown that the group 1 (PT1) contains 3364-bp (Ac superfamily transposon) at the promoter region of *Pi-ta*. It was also found that all of *Pi-ta* containing weedy accessions carries the same 3364-bp at the *Pi-ta* promoter region, whereas susceptible *pita* allele-carrying weedy accessions did not possess this fragment.

**Figure 3:**
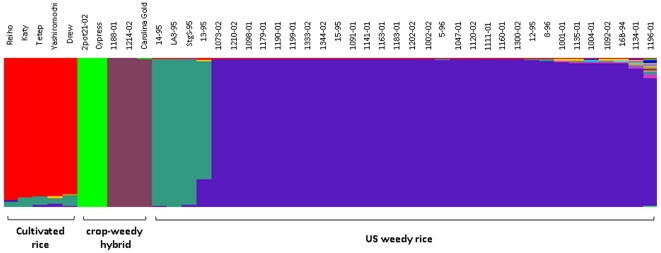
Structure diagram of rice accessions carrying *Pi-ta*. Name of accessions and presumed crop-weedy hybrid progeny were indicated.

The sequence at and around the region of the *Pi-ta* gene was found to be nearly identical among 34 weedy rice accessions and 10 cultivated rice (Kay, Drew, Madison, IR64, Tetep, Taducan, Teqing, Reiho, Pi-4, and Yashiro-Mochi) possessing *Pi-ta*. To determine the extent of sequence homology, SNP, Indels and SSR marker files on chromosome 12 in Group 1 accession were analyzed using STRUCTURE (Figure 3). This analysis placed Yashiro-Mochi, Reiho, Katy, Drew, and Tetep in the same genetic group, most distant from the predominant weedy rice accessions containing *Pi-ta*. This result indicates that *Pi-ta* in US weedy rice is genetically different from the common Asian and US cultivated rice. Four weedy rice accessions StgS-95, LA3-95, 13-95, and 14-95 were separated from the major weedy rice group, suggesting a different source of *Pi-ta* in these accessions.

### Identification of essential genomic region for blast resistance

**Figure 4:**
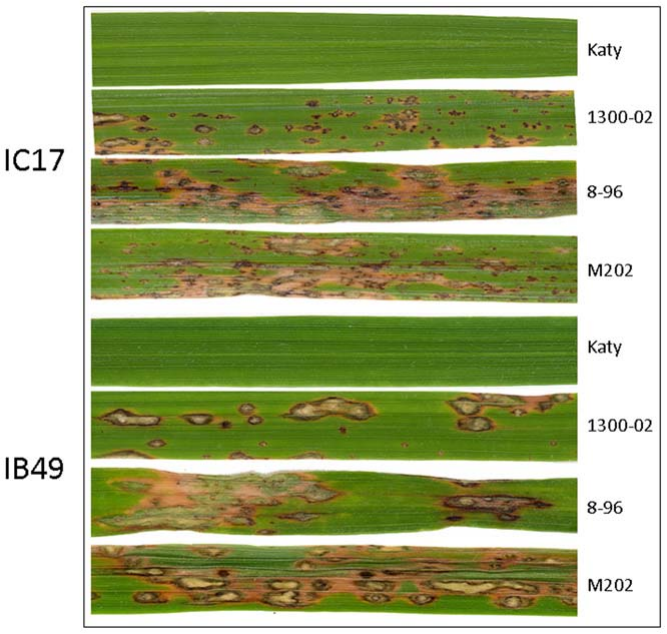
Blast symptoms of rice. Name of weedy rice and blast race were indicated. Pictures were scanned 7 days after inoculation.

To identify essential genomic regions involved in *Pi-ta* mediated blast resistance, we inoculated all 34 weedy rice accessions and a US cultivated rice Katy containing *Pi-ta* with two predominant US races of *M. oryzae* IB49 and IB17 (*AVR-Pita*). A high level of resistance was found in all accessions except three weedy rice accessions, 1111-01, 1300-02, and 8-96, which showed high degree of susceptibility to both pathogen races. Fifteen out of those 34 accessions showed the highest resistance to both blast races, similar to the resistance found in Katy, and the other 14 accessions were rated with disease resistance scores of 1 to 2 (Table 5). Two accessions, 1179-01 and 1141-01, showed moderated blast symptoms against both races. Two of the susceptible weed accessions, 1111-01 and 1300-02 were rated from 3 to 4. The accession 8-96 was the most susceptible to both races in all three replicated experiments (rating 4 for both blast races), which is comparable with the susceptible cultivar, M202 (Figure 4). We didn't find sequence polymorphism in the *Pi-ta* gene (including 3′ and 5′ regions) in 34 weedy accessions with other known cultivated rice containing *Pi-ta* (Kay, Drew, IR64, Tetep, Taducan, Teqing, Reiho, Pi-4, and Yashiro-Mochi). RT-PCR was used to determine if *Pi-ta* was expressed. The results showed that expression of *Pi-ta* in the susceptible accessions was similar to that in a resistant cultivar Katy (Figure S2). This analysis suggests that blast susceptibility was not due to the promoter mutations. We then examined if any sequence changes in the flanking region of the *Pi-ta* gene, which carries additional critical genes needed for resistance, in these susceptible accessions comparing with other resistant weedy accessions. Interestingly, the presence of nucleotide polymorphisms was first detected at the flanking gene sequence of OS12g20260 (11.8 Mb) suggesting that *Pi-ta* introgressed block was 2.2 Mb in these susceptible accessions. The size of the *Pi-ta* introgression block in resistant weeds and cultivars was at least 5 Mb suggesting that additional critical plant components needed for blast resistance may reside between 11.8 to 14.0 MB on chromosome 12.

**Table 5:** Disease reactions of US weedy rice accessions carrying the resistant *Pi-ta* allele.

Name of weedy rice accession and cultivars	Disease reaction^1^	
	IB49	IC17
LA3-95, 16B-94, 5-96, 1344-02, 14-95, 1134-01, 1092-02, 1135-01, 1091-01, 1073-02, 12-95, 1160-01, 1004-01, StgS-95, 1196-01	0–1	0–1
13-95, 1098-01, 1001-01, 1333-02, 1199-01, 1120-02, 1163-01, 1047-01, 15-95, 1210-02, 1002-02, 1202-2, 1190-01, 1183-01	1–2	1–2
1179-01, 1141-01	2–3	2–3
1111-01, 1300-02	3	3
8-96	4	4
Katy	0–1	0–1
M202	4	4

1 Blast reaction was evaluated using a 0 to 5 scale where 0 to 2 indicates resistant and 3 to 5 indicates susceptible respectively. Disease was evaluated at 7 days after inoculation using the method previously described [20].

### Gene flow at the *Pi-ta* locus

Analysis of 629 SNPs (*Pi-ta* gene and flanking region), one indel, and 13 SSRs on chromosome 12 revealed evidence of introgression at the susceptible *pi-ta* locus from US cultivars to weedy rice. The sequences of the *pi-ta* locus and six flanking gene fragments (1 Mb upstream and 1 Mb downstream of the *pi-ta* locus) were identical between two weedy accessions 1188-01 and 1214-02 and three cultivars Carolina Gold, Edith, and Blue Rose, which are early cultivars grown in the Southern US susceptible to major US races of *M. oryzae*. Thus, Carolina Gold was further selected for examining the introgression of *pi-ta* using SSR makers on chromosome 12. Structure analysis of combined SNP and SSR data showed that three accessions (1188-01, 1214-02, and Carolina Gold) were placed into one genetic cluster (Figure 4). Similarly, identical 2 Mb *pi-ta* regions were found in the weedy accession 2002-2-pot 21 and a popular US cultivar Cypress, which is also susceptible to major US races of *M. oryzae*. These results were in agreement with the phenotypic description of the parent plant of accession 2pot21-02 including a dark purple stem, smooth leaves, and no awns, in addition to typical black hull and red seeds; phenotypes that can indicate hybridization between red rice and cultivated rice (data not shown). These traits were also consistent with phenotypes that we found in segregating F_2_ progeny in crosses between ‘awned blackhull’ red rice and Cypress rice [8]. Taken together, we suggest that the identical *pi-ta* region may be shuffled among rice cultivars and weedy accessions. Evidence of gene flow of the *Pi-ta* gene between weedy rice accessions and deployed cultivars that contain *Pi-ta* in the US has not been found.

We then performed *F_st_* statistic analysis to determine extent of nucleotide divergence and distance among rice populations. From the *F_st_* statistics, US weedy accessions showed the least differentiation from the *indica* and greatest differentiation from *japonica* at the *Pi-ta* gene (Table 6). This pattern was consistent with the gene flow estimator, *Da*. SH accessions were more closely related to *indica* than any other accessions in the dataset. BHA accessions showed the least differentiation with *aus* and *aromatic*, while SH showed greatest differentiation with *aus* (Table 6). Two differentiation measures *F*_st_ and *D_a_* strongly indicate that US cultivars (*tropical japonica*) are very closely related to *japonica*, but greatly differentiated with SH weedy group.

**Table 6:** Number of net nucleotide divergence (*D_a_*) between populations (above diagonal) and *F_st_* genetic distance between groups (below diagonal) at the *Pi-ta* region^1^.

Population	US cultivar	All US weedy	SH	BHA	*aus*	*indica*	*japonica*	*aromatic*	*O. rufipogon*
***The Pi-ta*** **allele**									
US cultivar		0.00126	0.00203	0.00128	0.00158	0.00073	**0.00001**	0.00112	0.00056
All US weedy	0.39210		N/A^2^	N/A	**0.00048**	**0.00012**	0.00129	0.00027	0.00104
SH	0.61872	N/A		0.00091	0.00140	0.00057	0.00204	0.00120	0.00190
BHA	0.39408	N/A	0.53584		0.00005	0.00027	0.00135	**0.00001**	0.00092
*aus*	0.48584	0.28583	0.73857	**0.03841**		0.00053	0.00164	**0.00001**	0.00103
*indica*	0.23972	**0.06375**	**0.33434**	0.12891	**0.25530**		0.00079	0.00015	**0.00045**
*japonica*	**0.00001**	0.40912	0.63500	0.41710	0.50602	0.26160		0.00118	0.00063
*aromatic*	0.34328	0.14178	0.55608	0.00001	0.00001	0.06754	0.36346		0.00051
*O. rufipogon*	0.13376	0.25028	0.43951	0.22702	0.26523	**0.11384**	0.15202	0.13333	
**Flanking region**									
US cultivar		0.00058	0.00126	0.00057	0.00101	0.00033	**0.00001**	0.00144	0.00083
All US weedy	0.26655		N/A	N/A	0.00070	0.00038	0.00065	0.00126	0.00082
SH	0.56603	N/A		0.00112	0.00186	0.00129	0.00129	0.00186	0.00135
BHA	0.26855	N/A	0.57499		**0.00018**	**0.00021**	0.00070	**0.00018**	0.00101
*aus*	0.45350	0.38288	0.79039	0.14663		0.00041	0.00103	0.00028	0.00105
*indica*	0.12583	**0.14633**	**0.43234**	**0.09057**	**0.18519**		0.00044	0.00058	**0.00043**
*japonica*	**0.00001**	0.30113	0.59687	0.32647	0.48062	0.16726		0.00130	0.00070
*aromatic*	0.50520	0.48715	0.77392	0.41399	0.22857	0.22466	0.49757		0.00058
*O. rufipogon*	0.23946	0.24474	**0.41521**	0.28903	0.32842	**0.11769**	0.21609	0.19756	

1 The lowest *F_st_* values between populations are indicated in bold font. More than one value was bolded if the values are closely similar. Levels of genetic differentiation among population were estimated by *Da* and *F_st_* using DnaSP 5.10 (www.ub.es/dnasp). Total nine populations were compared with each other. The value close to zero means the least difference between populations. US cultivar (*tropical japonica*) accessions were not included in the *japonica* group.

2 Indicates not applied.

**Figure 5:**
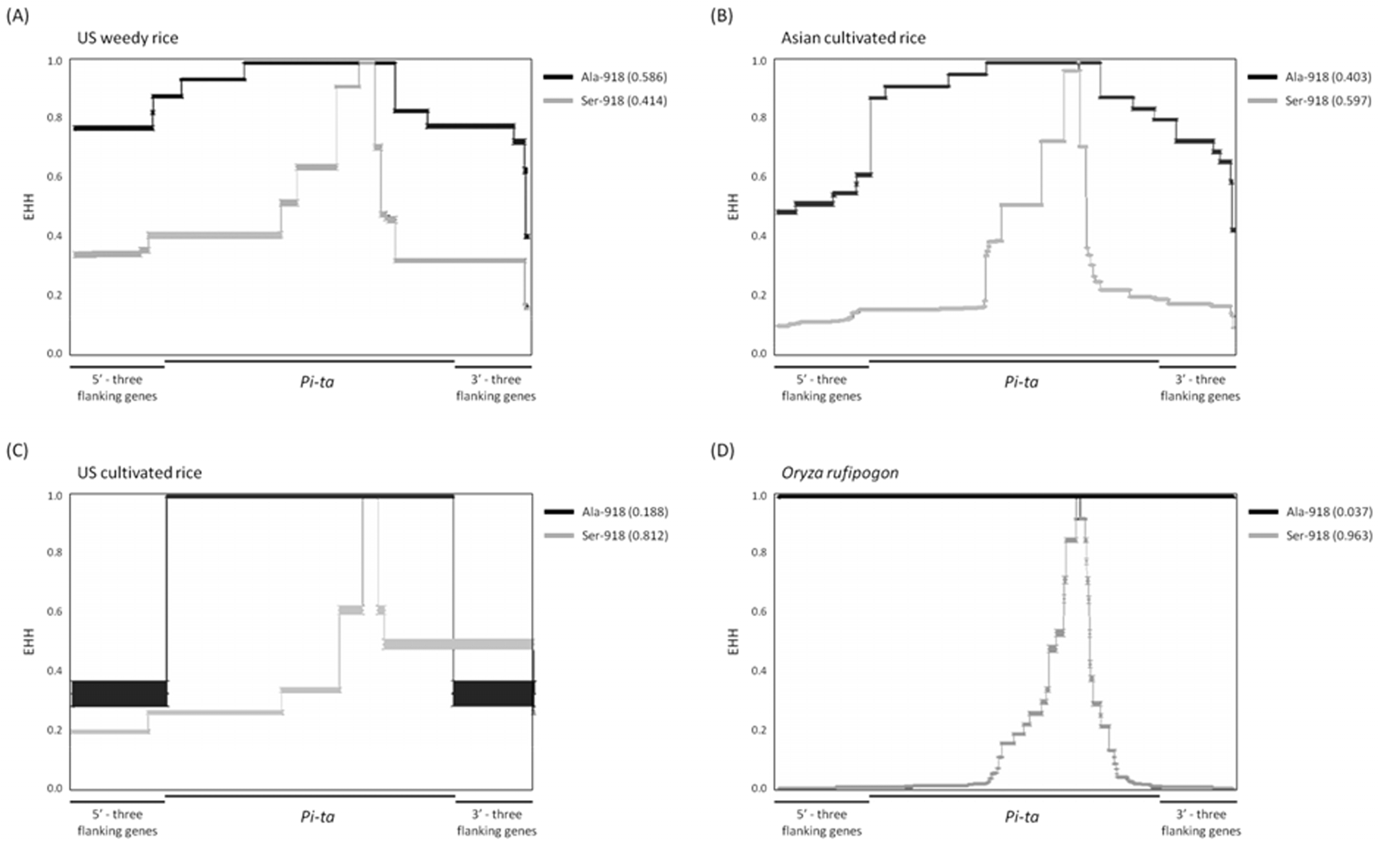
Extended haplotype homozygosity (EHH) at and around *Pi-ta*. The EHH of two core haplotypes (alanine-918 for resistant *Pi-ta* allele and serine-918 for representing susceptible *pi-ta* allele) were compared in the *Pi-ta* region of US weedy rice (A), Asian (B) and US cultivated rice (C), and wild species *O. rufipogon* (D).

Analysis of putative translated products revealed two major Pi-ta variants containing either Alanine or Serine at the position 918 in weedy rice populations. The extended haplotype homozygosity (EHH) analysis was then used to determine the extent of sequence diversity in these two haplotypes (Figure 4). More sequence diversity was detected around the haplotype Ser-918 than that with Ala-918 in all rice accessions. The patterns of EHH in US weedy rice were similar to that in Asian cultivated rice group containing *indica* and *aus*, but showed a slower degeneration of homozygosity for Pi-ta with Ala-918. In contrast, US cultivated rice group (*tropical japonica*) has very low EHH for Pi-ta with Ala-918 (0.188. In particular, US cultivated rice group showed high homozygosity at the *Pi-ta* locus and the breakdown of the homozygosity starts immediately in the flanking region, suggesting a simple source of *Pi-ta* was used for breeding. Not surprisingly, Ser-918 of *O. rufipogon* has the highest sequence variation (Figure 5). Please note that the EHH value for Ala-918 was near zero because only one accession of *O. rufipogon* possesses the Pi-ta variant with Ala-918 in this group (Fig. 5D). All patterns of EHH supports the hypothesis that Ala-918 (*Pi-ta*) in US weedy rice was recently derived from the ancestral *Pi-ta* allele carrying Ser-918 (*pi-ta*), and that it originated from Asian cultivated rice.

## Discussion

Weedy rice is one of the major problems in the rice growing areas of the southern US and it causes significant economic losses annually (Figure S3, Figure S4). Recently, genetic diversity of a few important agronomic traits in US weedy rice have been investigated [2], [3], [5], [6]. In the present study, using a large sequence data set of the *Pi-ta* gene and its flanking region, we determined the genome organizations and origin of the *Pi-ta* gene in US weedy rice. We found that nucleotide diversity at and around the *Pi-ta* locus in US weedy rice is exceptionally low when compared with that in other *Oryza* species. This result indicates the occurrence of genetic bottlenecks during the establishment of weedy rice in the US that is also consistent with a few other reports [2], [3], [5], [6]. It has been known that LRD of the *Pi-ta* coding region is functionally conserved in domesticated and wild rice [22], [23]. We found that LRD region in US weedy rice is also under purifying selection. As described in previous studies, the *Pi-ta* gene has been evolved under recent directional selection in *O. rufipogon* [22], [23]. Interestingly, directional selection is also found in SH weedy groups possessing *Pi-ta* in the present study. These findings suggest that *Pi-ta* in US weedy rice has been recently derived from the ancestral origin, and has been evolving in the US weedy rice populations.

We have not found any evidence to say that the gene flow of *Pi-ta* has occurred between cultivated US rice cultivars and weedy rice in the US. The *Pi-ta* gene in US cultivars was initially bred from Tetep [15]. If the *Pi-ta* gene in weedy rice in the US is not from deployed cultivars an alternative origin of the *Pi-ta* gene should exist. Phylogenic analyses revealed that the *Pi-ta* gene in weedy red rice was more similar to *aus*, *aromatic* landraces, and Asian *indica* (or *japonica* containing the *indica*-derived *Pi-ta*) (Figure 2). The pattern of genomic diversity and population differentiation at/around the *Pi-ta* gene in all US weedy rice revealed that the *Pi-ta* locus in weedy rice was genetically more similar to that in Asian cultivated rice (*indica*) rather than US cultivars and *O. rufipogon*, respectively. US weedy accessions can be divided into two major subgroups, SH and BHA. Our sequence and marker data demonstrated that SH is similar to *indica* and BHA is similar with *aus*. These findings suggest that *Pi-ta* in weedy rice could be derived from Asian rice distinct from those used to introgress *Pi-ta* into US germplasm. In plants, some resistance genes show gene presence/absence polymorphisms (P/A). These P/A polymorphisms can be selectively maintained for long evolutionary periods with flanking regions bearing the molecular signatures of balancing selection [27]. We have identified a P/A polymorphism at 2 Mb downstream from *Pi-ta*. This polymorphism was used to understand the origin of the *Pi-ta* gene in US red rice because this P/A polymorphism was found in majority of weedy accessions (48 of the 50 accessions tested). The same polymorphism was found in two Asian cultivars Yashiro-mochi and Te Qing and *O. rufipogon.* This result further supports that *Pi-ta* in US red rice may have originated from Asian cultivars or/and *O. rufipogon* rather than from US cultivars.

Selective introgression of resistance genes has been an effective method in breeding blast resistant rice. However, the escape of deployed resistance genes to weedy relatives may create new problems, and this has never been examined in the rice-farming ecosystem. Recent studies have shown that hybridization events have occurred between cultivated and weedy red rice [4], [5], [12], [28], [29], [30]. The gene flow frequency between cultivated rice and weedy rice was estimated to be between 0.011 and 0.046 and can be up to 1% [10], [31]. This rate of gene flow can potentially bring costly consequences to the advanced breeding of rice blast resistance [12]. In the present study, we found two lines of evidence that gene flow may occur at the susceptible *pi-ta-*carrying locus from US cultivars to weed accessions 1188-01, 1214-02, and 2pot21-02. First, the identical *pi-ta* genomic region was found in weedy accessions 1188-01 and 1214-02 and US cultivars Carolina Gold. These weedy accessions were also collected from East Carroll Parish, Louisiana where the cultivar was once grown. In a previous study by Reagon *et al*. [5], there was no evidence of introgression in these two weedy accessions. However, in the present study, we detected no sequence polymorphism at/around the *pi-ta* gene (∼2Mb) among 1188-01 and 1214-02 with Carolina Gold, Edith, and Blue Rose, that were once the leading US varieties in the South from 1910 to 1945. We have also shown a nearly identical SSR genotype in these two weedy accessions with Carolina Gold, indicating that the allele in the weedy rice may be due to shared ancestry. Secondly, accession 2002-02-pot21 was previously determined to be an admixture through hybridization using 48 sequence STS makers [5]. In the present study, we determined that 2pot21-02 collected from Lawrence County, AR, where Cypress once was grown, possesses the same *pi-ta* genomic region of the US cultivar Cypress. The susceptible *pi-ta* region in the weedy accessions of presumed crop-weed progenies was genetically identical to that in US cultivars, while other weedy accessions were genetically more similar to *indica*. However, direct evidence of gene flow of *Pi-ta* from cultivated to weedy rice has not been found in this study. A much larger number of weedy rice samples would be required for the verification of this phenomenon.

In the present study, we demonstrated that most weedy rice accessions containing *Pi-ta* were highly resistance to the predominant US blast races IB49 and IC17. Among 34 weedy rice accessions possessing *Pi-ta*, three weedy accessions 1111-01, 8-96, and 1300-02 showed susceptibility to IB49 and IC17. After analyzing sequences at and around the *Pi-ta* locus, we found that the susceptible weedy accessions have a shorter size of *Pi-ta* introgression (2.2 Mb) than that in other resistant weedy and cultivated accessions (at least 5 Mb). It was recently reported that the size of *Pi-ta* introgression block is closely associated with the resistance specificity to races of blast pathogen [13], [23]. Jia [13] recently reported that the largest linkage block of *Pi-ta* was identified in backcrossing and elite rice cultivars. A rice gene, *Ptr(t)* required for the *Pi-ta*-mediated resistance, was recently mapped within a 9 Mb region spanning the *Pi-ta* gene [25], [17]. *Ptr(t)* may reside within an 2.8 Mb genomic region beyond 11.8 MB on chromosome 12 because three weedy accessions without this region were susceptible to blast fungus.

In summary, we have demonstrated that genetic exchange can occur at a locus that is involved in blast disease resistance. Despite the widespread deployment of *Pi-ta* over two decades in the US, introduction of a *Pi-ta* gene in weedy rice and impact on blast management in weedy rice has not yet been characterized. Further studies of susceptible weedy accessions with *Pi-ta* should help to develop effective strategies for managing rice blast disease and weedy species of rice.

## Materials and Methods

### Plant materials and DNA sequencing

A total of 58 US weedy rice accessions were collected from1994 to 2004 from rice fields in the Southern USA (Table S1). Those accessions were subsequently propagated via single seed descent in field plots at the USDA Dale Bumpers National Rice Research Center (DB NRRC). The weedy rice accessions consisted of four different groups, SH, BHA, BR, and crop-weed hybrids (MIX) [5]. Among them, SH and BHA were the most common ecotypes (Table S1). BR weedy ecotypes were hybrids of BHA and SH strains [5]. The rice accessions studied in Lee *et al* [23] from cultivated and wild *Oryza* species were included in the present study: *O. sativa* (38 Asian and 16 US cultivated varieties), the crop's wild progenitor *O. rufipogon* (28 geographically diverse accessions, two accessions of *O. nivara*), and accessions of other AA genome *Oryza* species *O. meridionalis* (2), *O. glumaepatula* (2), *O. glaberrima* (4) and *O. barthii* (2) (Table S2). The same primers and sequence methods were used as in a previous study [20]. Sequences of the *Pi-ta* gene for all accessions used in this study are from GenBank accession GQ918334-GQ918489. Plants were grown in the greenhouses at Washington University, University of Massachusetts, and in DB NRRC for DNA extraction.

DNA was extracted using a modified cetyltrimethylammonium bromide (CTAB) method. Fifteen primer pairs were designed using the Primer3 program to amplify the *Pi-ta* genomic region including promoter and downstream sequences. All developed primers were compared by BLAST to the *indica* (93-11) and *japonica* (Nipponbare) reference genomes to ensure their target specificity. A fragment of 7252 bp of the *Pi-ta* locus including the intron and 5′ leader and 3′ trailer region, and 12 gene fragments spanning the *Pi-ta* gene within 8 megabases were sequenced.

### Evaluation of disease reaction and expression of *Pi-ta*

Two avirulent US races of *M. oryzae*, IB49 (ZN61) and IC17 (ZN57) containing *AVR-Pita1* were used to inoculate US weedy rice that contained *Pi-ta* [16]. Weedy rice was planted in three replicate pots with three to four seedlings per pot and grown in a greenhouse. A highly resistant (Katy with *Pi-ta*) and susceptible (M202 without *Pi-ta*) rice varieties were used as positive and negative controls, respectively. Inoculation and disease assays were performed following the methods as described [23]. Disease reactions were rated on a semi-quantitative scale one week after inoculation as 0 to 2 for resistance and 3 to 5 for susceptibility.

RT-PCR was performed to examine the expression of *Pi-ta* in the three US weedy rice accessions, 1111-01, 1300-02, and 8-96, that contained the resistant *Pi-ta* allele, but were susceptible to IB49 and IC17, US cultivar Katy carrying *Pi-ta* was used as a positive control. A pair of primers (KG2: ATCAGCAACTAACGAGGCAT and YL88: TACAGGTTCAATTTCTGTTG) [25] were used for examining expression of *Pi-ta*.

### DNA marker analysis

To determine the chromosome-wide introgression patterns around the *Pi-ta* locus, a total of 14 DNA markers including one SNLP (*Pita*) [18] and 13 Simple Sequence Repeat (SSR) markers (RM3483, RM5746, RM7003, RM3246, RM27941, XY121, XY196, RM27946, RM27973, RM7102, RM511, RM463, and RM1300; physical location from 1.61 Mb to 25.97 Mb) evenly distributed across rice chromosome 12 were analyzed in 36 US weedy rice and seven cultivated rice. The sequence for all SSR markers except for XW121 (F:CGACAGGAAAACTTGTTAGGAA; R: AGTTGTGTGTCGCTTGCTGT) and XW196 (F:TGTCATTAGCAGCTACGGTGGT and R: TGTTTGACCGTGGTCTTGCT) can be located at www.gramene.org.

### DNA Sequence analysis

Sequences were aligned and edited using the software DNASTAR, Lasergene 8 and Mega 4. The patterns of nucleotide polymorphisms and molecular evolution at and around the *Pi-ta* region were analyzed using DnaSP 4.9 [32]. Average pairwise nucleotide diversity (θ_π_) and Watterson's estimator (θ*_w_*) were calculated for the *Pi-ta* locus and flanking gene fragments in US weedy rice, other groups of *O. sativa*, and *O. rufipogon*. Phylogenetic analysis of the *Pi-ta* locus in rice accessions was performed using genetic distance-based clustering algorithms (Neighbor-joining). The tree was constructed based on 11 Kb DNA sequences including six flanking genes near the *Pi-ta* locus covering 2 Mb of the *Pi-ta* region in rice. The extent of linkage disequilibrium and the extended haplotype homozygosity (EHH) was used to assess how selection and introgression have shaped the molecular evolution of the *Pi-ta* gene in US weedy rice. EHH across the sampled genomic region containing the *Pi-ta* gene was calculated to visualize the effect of selection on the Ala-918 and Ser-918 containing alleles as described [33], [34], [35]. The possibility of selection on the *Pi-ta* gene and on flanking genomic fragments was examined with Tajima's *D* using MEGA 5 [36]. The software STRUCTURE [37], [38], [39] was used to compare the population structure of the *Pi-ta* genomic region in US weedy rice with cultivated rice and identify migrant or admixed accessions. A total of 69 SNPs from the *Pi-ta* region, one insertion/deletion (*indel*) and 13 SSR marker genotypes from rice chromosome 12 were used for analysis. All STRUCTURE analyses were performed using the model of admixture with allele frequencies correlated for *K* (number of clusters) = 1−20 and every run was repeated 10 times to obtain the run with the highest penalized log-likelihood score. The analysis was repeated 50000 times (10000 burn-in periods) on random subsets of the data for a range of different numbers of loci.

## Supporting Information

Figure S1 **DNA sequence polymorphism at the** ***Pi-ta*** **gene in US weedy rice.** Sliding window analysis of 7275 DNA sequences of the *Pi-ta* gene analyzed using DNASP software. Sites with alignment gaps were not counted in the window length (and slide). Window length was 50 and step size was 10. Graphic presentation of *Pi-ta* was shown at the bottom.
(TIF)

Figure S2 **Expression of the** ***Pi-ta*** **gene in US weedy rice accessions and cultivar Katy containing** ***Pi-ta*** **.** Lane 1 Accession, 1111-01, lane 2, 1300-02, lane 3, 8-96, lane 4, Katy, and 5: Katy genomic DNA as a negative control.
(TIF)

Figure S3 **Photograph of rice seeds of a US cultivar and a red rice.** Seeds with/without hull were shown.
(TIF)

Figure S4 **Weedy rice found in a commercial rice field, Stuttgart AR.** Most of rice plants shown in the photo are weedy rice. Over 70% of field area was contaminated by the weedy rice population.
(JPG)

Table S1 **Rice accessions of seven AA genome** ***Oryza*** **species used in the present study.**
(DOC)

Table S2 **Description of the US weedy rice accessions used in present study.**
(DOC)
