# Supplementary figures and images for: Molecular Evolution of the Rice Blast Resistance Gene Pi-ta in Invasive Weedy Rice in the USA
Source: PLoS One. 2011 Oct 17;6(10):e26260. doi: 10.1371/journal.pone.0026260 (PMC3197024; doi:10.1371/journal.pone.0026260)

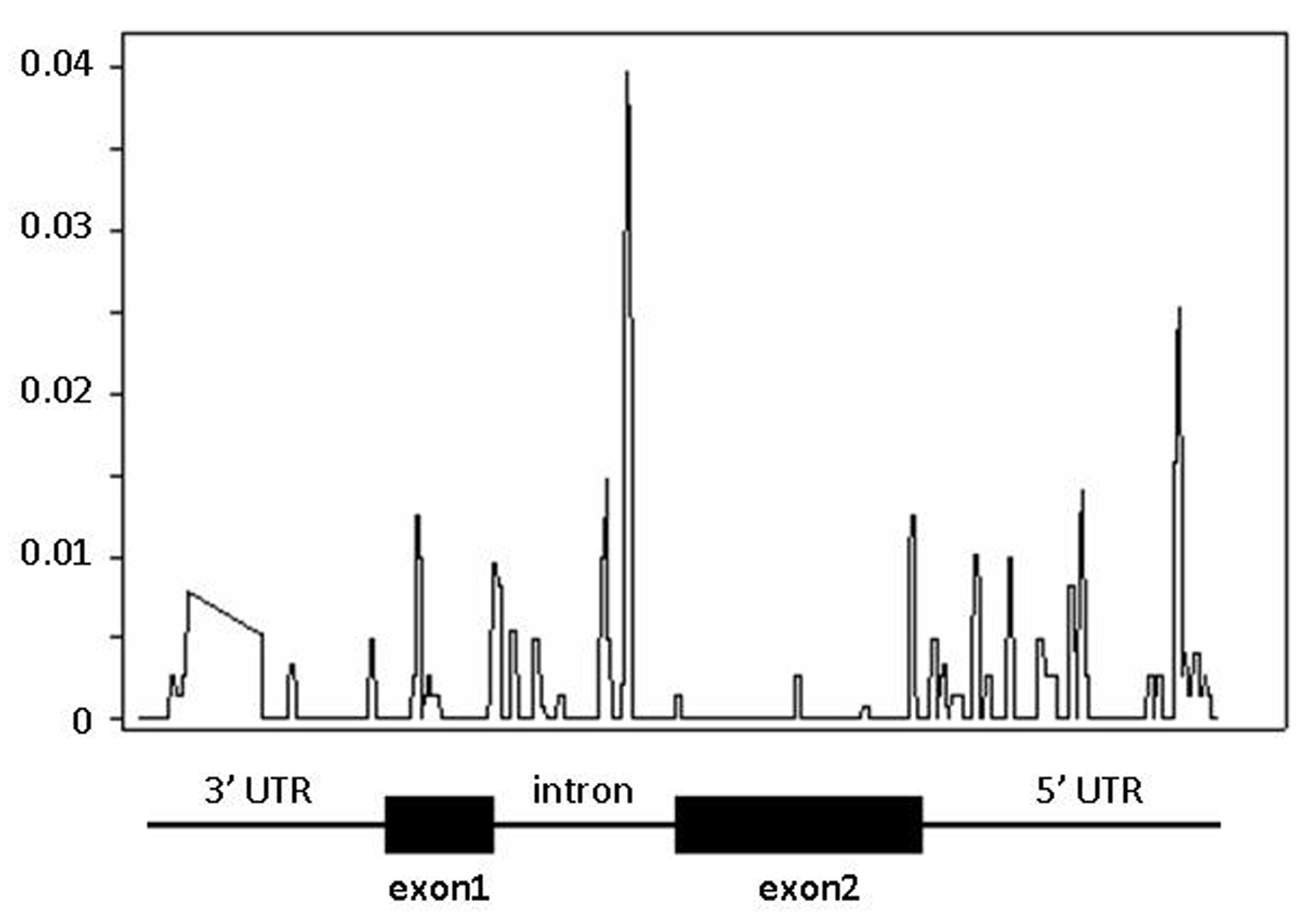

Supplement: Figure S1 — DNA sequence polymorphism at the Pi-ta gene in US weedy rice. Sliding window analysis of 7275 DNA sequences of the Pi-ta gene analyzed using DNASP software. Sites with alignment gaps were not counted in the window length (and slide). Window length was 50 and step size was 10. Graphic presentation of Pi-ta was shown at the bottom. (TIF) [file pone.0026260.s001.tif]

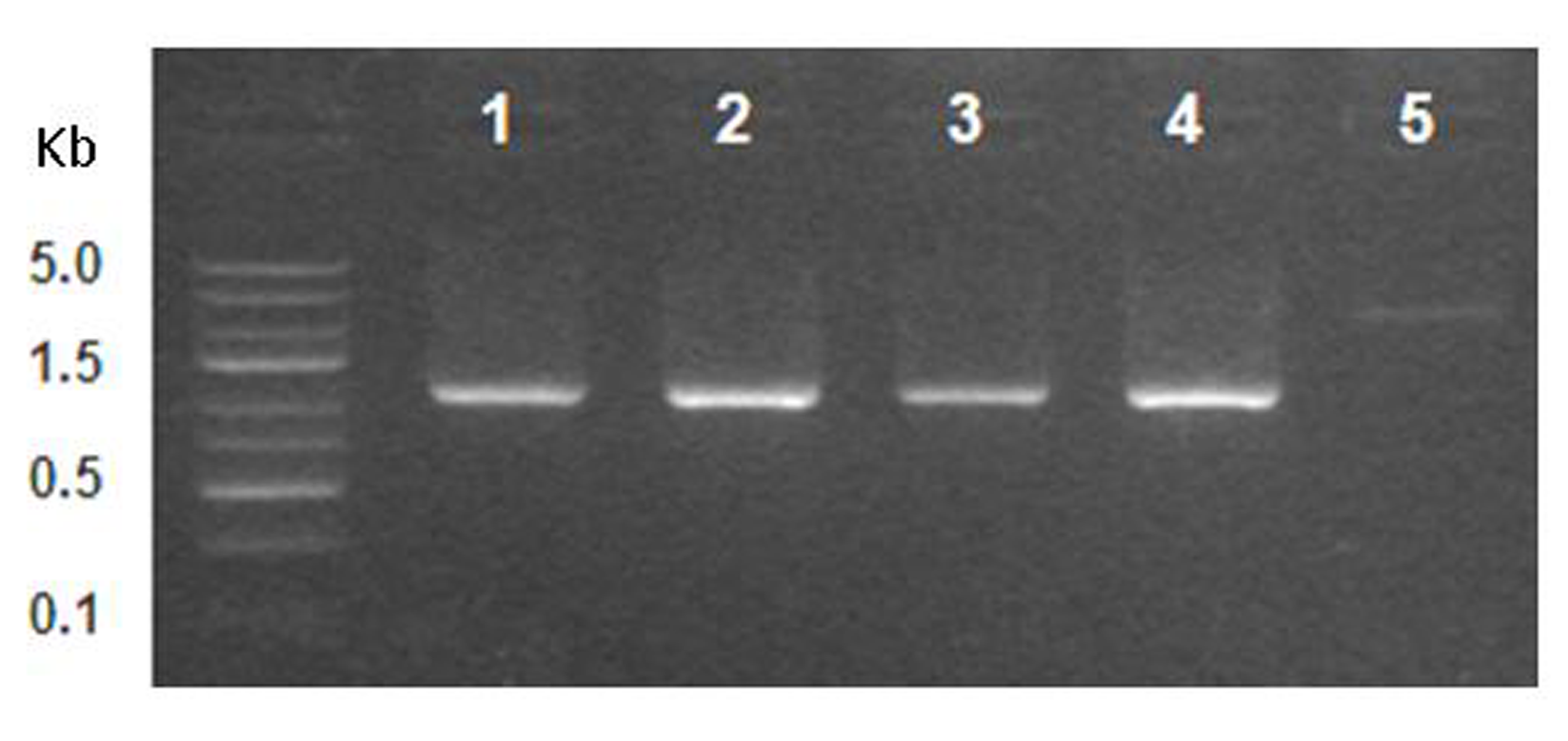

Supplement: Figure S2 — Expression of the Pi-ta gene in US weedy rice accessions and cultivar Katy containing Pi-ta . Lane 1 Accession, 1111-01, lane 2, 1300-02, lane 3, 8-96, lane 4, Katy, and 5: Katy genomic DNA as a negative control. (TIF) [file pone.0026260.s002.tif]

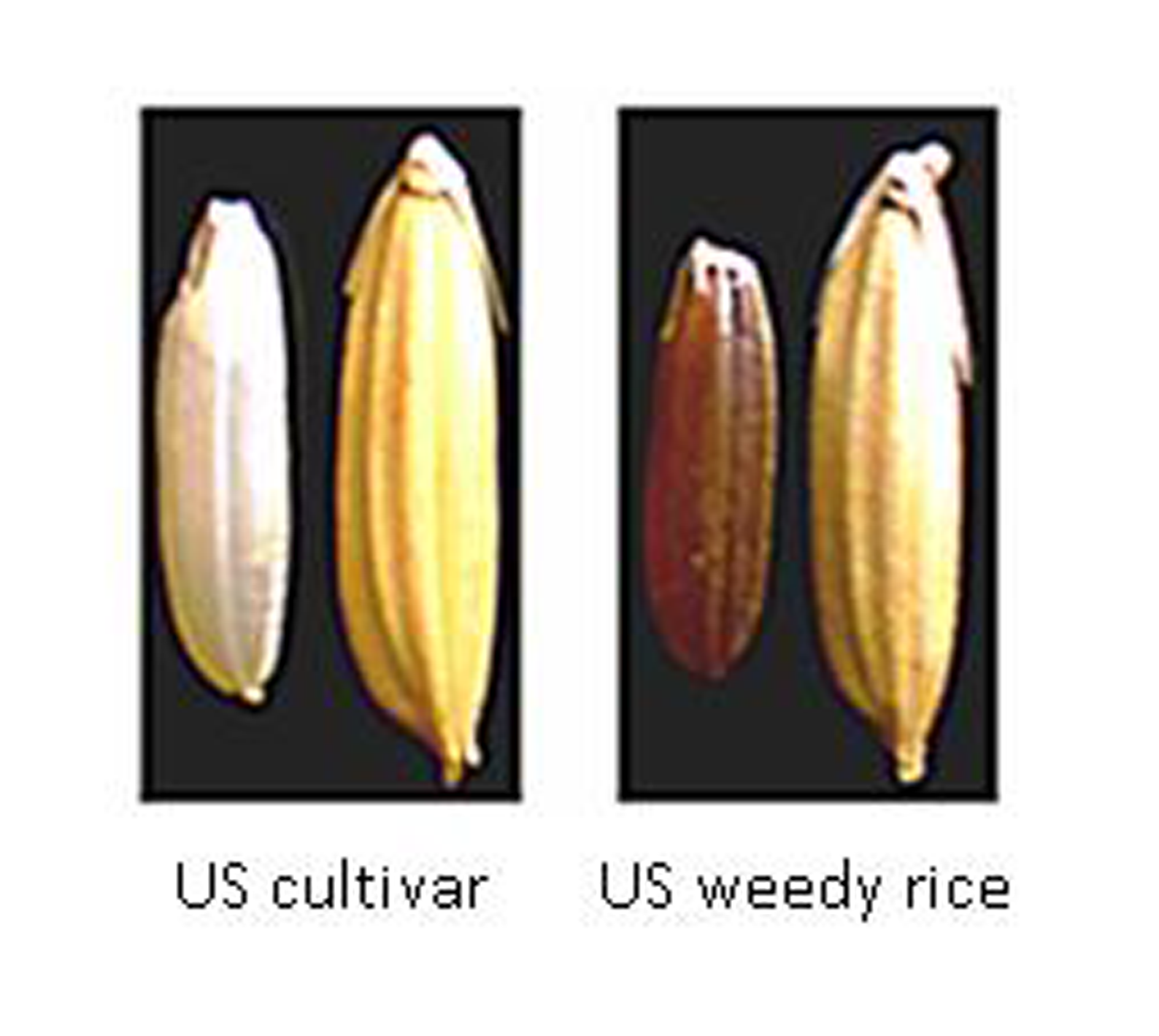

Supplement: Figure S3 — Photograph of rice seeds of a US cultivar and a red rice. Seeds with/without hull were shown. (TIF) [file pone.0026260.s003.tif]

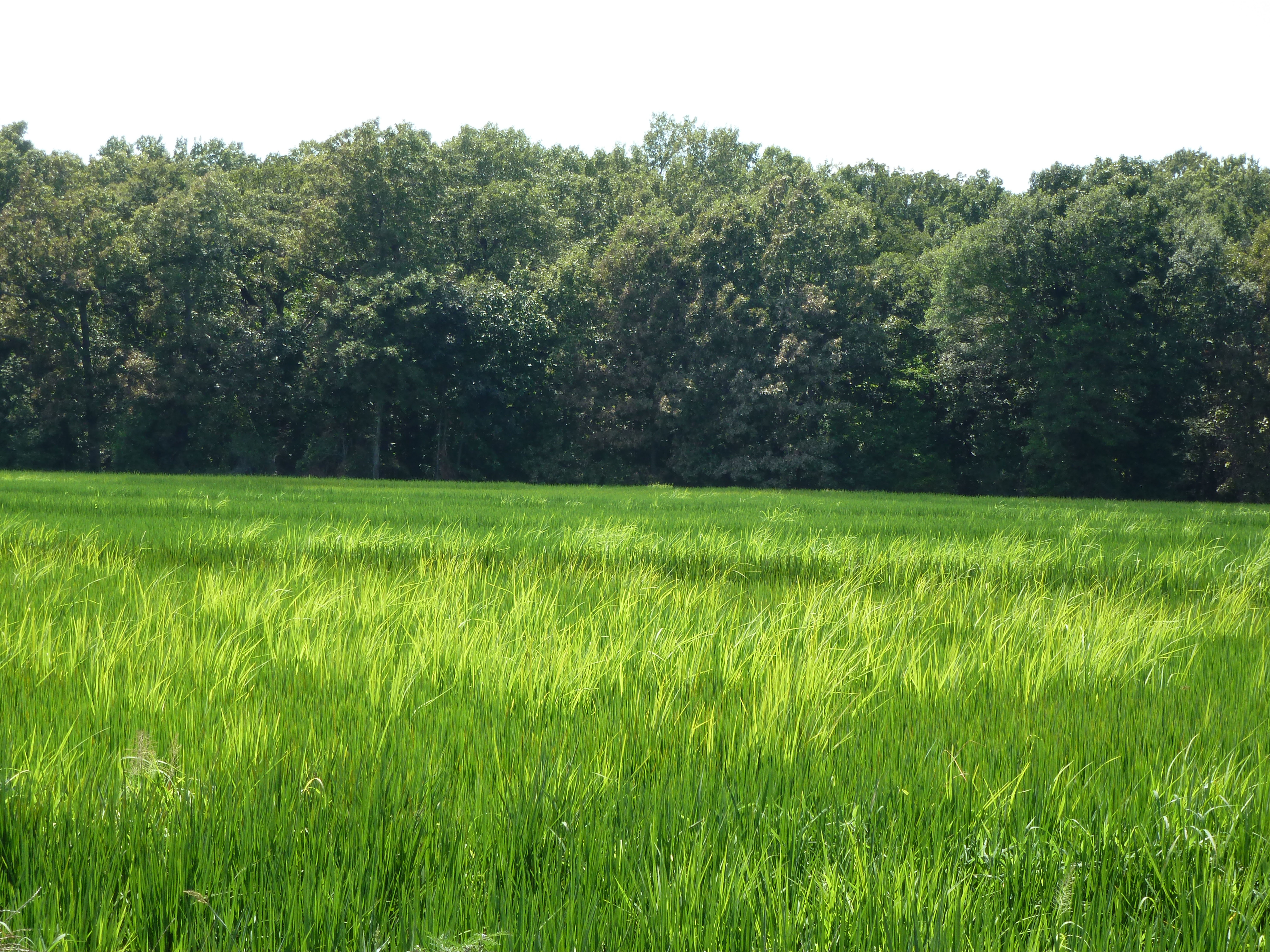

Supplement: Figure S4 — Weedy rice found in a commercial rice field, Stuttgart AR. Most of rice plants shown in the photo are weedy rice. Over 70% of field area was contaminated by the weedy rice population. (JPG) [file pone.0026260.s004.jpg]
