# Supplementary material for: Molecular Evolution of the Rice Blast Resistance Gene Pi-ta in Invasive Weedy Rice in the USA
Source: PLoS One. 2011 Oct 17;6(10):e26260. doi: 10.1371/journal.pone.0026260 (PMC3197024; doi:10.1371/journal.pone.0026260)
Supplement: Table S1 — Rice accessions of seven AA genome Oryza species used in the present study. (DOC) [file pone.0026260.s005.doc]

**Table S1.** Description of the US weedy rice accessions used in the present study.

| **Accession^a^** | **Accession abbreviation^b^** | **Type**^c^ | **Origin** | **Group**^d^ |
| --- | --- | --- | --- | --- |
| MO-2001-1004 [PI 653426] | 1004-01 | SH | Dunklin County, MO | 1 |
| AR-2001-1025 [PI 653427] | 1025-01 | BHA | Clay County, AR | 3 |
| AR-2001-1081 [PI 653428] | 1081-01 | BHA | Perry County, AR | 2 |
| AR-2001-1091 [PI 653429] | 1091-01 | SH | Poinsett County, AR | 1 |
| AR-2001-1096 [PI 653430] | 1096-01 | BHA | Arkansas County, AR | 2 |
| AR-2001-1196 [PI 653431] | 1196-01 | SH | Crittenden County, AR | 1 |
| MO-2001-1098 [PI 653432] | 1098-01 | SH | Bollinger County, MO | 1 |
| AR-2001-1134 [P653434] | 1134-01 | SH | Lee County, AR | 1 |
| AR-2001-1135 [PI 653435] | 1135-01 | SH | Desha County, AR | 1 |
| AR-2001-1141 [PI 653436] | 1141-01 | SH | Lawrence County, AR | 1 |
| LA-2001-1160 [PI 653437] | 1160-01 | SH | Morehouse Parish, LA | 1 |
| MS-2001-1179 [PI 653438] | 1179-01 | SH | Coahoma County, MS | 1 |
| LA-2001-1188 [PI 653439] | 1188-01 | BHA | East Carroll Parish, LA | 3 |
| AR-1994-10A [PI 653413] | 10A-94 | BHA | Prairie County, AR | 2 |
| AR-1994-16B [PI 653414] | 16B-94 | SH | Prairie County, AR | 1 |
| AR-1994-18A [PI 653415] | 18A-94 | BHA | Stuttgart, AR | 2 |
| MS-1995-15 [PI 653416] | 15-95 | SH | Shaw, MS | 1 |
| AR-1996-1 | 1-96 | MIX | Arkansas County, AR | 4 |
| MS-1996-5 [PI 653418] | 5-96 | SH | Mississippi, MS | 1 |
| MS-1996-9 [PI 653419] | 9-96 | BHA | Mississippi, MS | 2 |
| LA-1995-LA3 [PI653420] | LA3-95 | BHA | Crowley, LA | 1 |
| MS-1995-MS4 [PI653421] | MS4-95 | MIX | Mississippi, MS | 2 |
| AR-1995-StgB [PI 653422] | StgB-95 | BHA | Stuttgart, AR | 2 |
| AR-1995-StgS [PI 653423] | StgS-95 | BHA | Stuttgart, AR | 1 |
| TX-1995-TX4 [PI653424] | TX4-95 | BHA | Katy, TX | 3 |
| AR-2001-1001 | 1001-01 | SH | Cross County, AR | 1 |
| AR-2002-1002 | 1002-02 | SH | Independence County, AR | 1 |
| AR-2002-1005 | 1005-02 | BHA | Faulkner County, AR | 2 |
| AR-2001-1042 | 1042-01 | BHA | Jefferson County, AR | 2 |
| LA-2001-1047 | 1047-01 | SH | Morehouse Parish, LA | 1 |
| MO-2002-1073 | 1073-02 | SH | Butler County, MO | 1 |
| MS-2002-1092 | 1092-02 | BR | Coahoma County, MS | 1 |
| AR-2001-1111 | 1111-01 | SH | Woodruff County, AR | 1 |
| LA-2001-1190 | 1190-01 | SH | East Carroll Parish, LA | 1 |
| MO-2001-1199 | 1199-01 | SH | Ripley County, MO | 1 |
| MO-2002-1300 | 1300-02 | BR | Dunklin County, MO | 1 |
| MO-2002-1344 | 1344-02 | SH | Stoddard County, MO | 1 |
| AR-PrairieCoShort_8 | PrairieCoShort8 | BHA | Prairie County, AR | 2 |
| AR-PrairieCoTall_10 | PrairieCoTall10 | BHA | Prairie County, AR | 2 |
| AR-PrairieCoTall_11 | PrairieCoTall11 | BHA | Prairie County, AR | 2 |
| AR-PrairieCoTall_17 | PrairieCoTall17 | BHA | Prairie County, AR | 2 |
| LA-1995-12 | 12-95 | SH | Crowley, LA, | 1 |
| LA-1995-13 | 13-95 | BHA | Crowley, LA, | 1 |
| LA-1995-14 | 14-95 | BHA | Crowley, LA, | 1 |
| MS-1996-8 | 8-96 | SH | Mississippi, MS | 1 |
| 2002-51 | 51-02 | MIX | - | 4 |
| AR-2004-1A | 1A-04 | MIX | Amagon, AR | 5 |
| AR-2001-1183 | 1183-01 | SH | ChiCot County, AR | 1 |
| MS-2002-1166 | 1166-02 | BHA | Coahoma County, MS | 2 |
| AR-2001-1107 | 1107-01 | BHA | Cross County, AR, | 2 |
| MO-2002-1210 | 1210-02 | SH | Dunklin County, MO | 1 |
| LA-2002-1214 | 1214-02 | BHA | East Carroll Parish, LA | 3 |
| MO-2001-1163 | 1163-01 | SH | Morehouse County, MO | 1 |
| AR-2002-1120 | 1120-02 | BR | St. Francis County, AR | 1 |
| MO-2002-1333 | 1333-02 | SH | Stoddard County, MO | 1 |
| AR-2002-1202 | 1202-02 | BHA | Jefferson County, AR | 1 |
| AR-2002-2-pot1 | 2pot1-02 | BHA | Jackson County, AR | 2 |
| AR-2002-2-pot21 | 2pot21-02 | MIX | Lawrence County, AR | 5 |

^a^ USDA-ARS DBNRRC red rice accession numbers (Gealy et al., 2009) and PI numbers in Germplasm Resources Information Network (GRIN: http://www.ars-grin.gov/npgs/) when available.

^b^ Accession abbreviation: code number-year designation (e.g. abbreviation for ‘MO-2001-1004’ = ‘1004-01’).

^c^ SH: Strawhull awnless, BHA: Blackhull awned, BR: Brownhull awned, MIX: presumed crop-weed hybrid progenies; these designations were derived from the population structure analysis of US weedy rice using genome-wide STS marker (Reagon et al., 2010).

^d^ Group name for weedy rice accessions were described in Figure 1.
