# Supplementary material for: Molecular Evolution of the Rice Blast Resistance Gene Pi-ta in Invasive Weedy Rice in the USA
Source: PLoS One. 2011 Oct 17;6(10):e26260. doi: 10.1371/journal.pone.0026260 (PMC3197024; doi:10.1371/journal.pone.0026260)
Supplement: Table S2 — Description of the US weedy rice accessions used in present study. (DOC) [file pone.0026260.s006.doc]

**Table S2.** Rice accessions of seven AA genome *Oryza* species used in the present study.

| **Species** | **No. of accession** | **Name and total number of geographic origins** |
| --- | --- | --- |
| *O. sativa* (Asian) | 38 | Bangladesh, China, India, Sri Lanka, Japan, Thailand, Vietnam, Iran, Indonesia, South Korea, Nepal, Philippines |
| *O. sativa* (Weedy rice) | 58 | USA |
| *O. sativa* (US variety) | 16 | USA |
| *O. rufipogon* | 28 | Bangladesh, China, Cambodia, India, Indonesia, Laos, Myanmar, Papau New Guinea, Thailand, Vietnam, Malaysia, Philippines, Sri Lanka, Taiwan |
| *O. barthii* | 2 | Nigeria, Mali |
| *O. glaberrima* | 4 | Niberia, Nigeria, Mali, Burkina Faso |
| *O. glumaepatula* | 2 | Colombia, Brazil |
| *O. meridionalis* | 2 | Australia, Indonesia |
| *O. nivara* | 2 | China, Nepal |
| **Total** | **152** | **26** |
